# Bacteriology of the burn wound at the Bai Jerbai Wadia Hospital for children, Mumbai, India—A 13-year study, Part I-Bacteriological profile

**DOI:** 10.4103/0970-0358.59284

**Published:** 2009

**Authors:** Shankar Srinivasan, Arvind M. Vartak, Aakanksha Patil, Jovita Saldanha

**Affiliations:** Department of Burns and Plastic Surgery, B.J. Wadia Hospital for Children, Parel, Mumbai, India

**Keywords:** Bacteriology in burns, burn wounds, paediatric burns

## Abstract

**Aim:**

To study which organisms were prevalent in our burn unit and their antibiotic sensitivity pattern in brief.

**Method:**

Microbiological data of 1534 patients admitted to the burns unit of the Bai Jerbai Wadia Hospital for Children, Mumbai over a period of 13 years (1994-2006) was reviewed retrospectively. A total of 9333 swabs were cultured and antibiotic sensitivities to the isolated organisms determined. The age group of patients admitted to our facility ranged from one month to 15 years.

**Result:**

Klebsiella was the predominant organism in our set-up (33.91%), closely followed by Pseudomonas (31.84%). The antibiotic sensitivities of the isolated organisms are discussed in detail in the text.

**Conclusion:**

Every treatment facility has microorganisms unique to it and these change with time. It is therefore of paramount importance to have an in-depth knowledge of the resident organisms and their antibiotic sensitivity pattern so that infection-related morbidity and mortality are improved.

## INTRODUCTION

Burn injury is a major problem in many parts of the world. It has been estimated that 75% of all deaths following burns are related to infection. Thermal injury destroys the skin barrier that normally prevents invasion by microorganisms. This makes the burn wound the most frequent origin of sepsis in these patients.[1]

Initially, the burnt area is considered free of microbial contamination. But gram-positive bacteria in the depth of sweat glands and hair follicles heavily colonize the wounds within 48 h of the injury.[2,3]

Topical antimicrobials decrease microbial overgrowth but seldom prevent further colonization with other potentially invasive bacteria and fungi. These are derived from the patient's gastrointestinal and upper respiratory tract and the hospital environment.[4,5]

Following colonization, these organisms start penetrating the viable tissue depending on their invasive capacity, local wound factors and the degree of the patient's immunosuppression.[5] If sub-eschar tissue is invaded, disseminated infection is likely to occur.[3] Great emphasis must therefore be placed on early identification of local signs of invasive burn wound infection.

The causative infective microorganisms in any burn facility change with time.[6,7] Individual organisms are brought into the burns ward on the wounds of new patients. These organisms then persist in the resident flora of the burn treatment facility for a variable period of time, only to be replaced by newly arriving microorganisms. Introduction of new topical agents and systemic antibiotics influence the flora of the wound.[6,7]

Thus, it is just not sufficient to be aware of the microorganisms that pose a problem for burn patients. To have an in-depth knowledge of the organisms that are predominant in that particular treatment facility during the particular period along with their sensitivity pattern is vital as many septic burn patients need to be treated with antibiotics before the results of microbiological cultures are available. This would be crucial to reduce the overall infection-related morbidity and mortality.

In the present study, we determined the nature of microbial wound colonization in 1534 patients. The major objectives were to determine:

- Which microorganisms were prevalent in our treatment facility,
- Their antibiotic sensitivity pattern.

## MATERIAL AND METHODS

### Patients

This is a retrospective analysis of the study of isolates from the burns unit of Bai Jerbai Wadia Hospital for children, Mumbai. The hospital caters exclusively to a paediatric population. In our study, the youngest child was a month old and the oldest, 15 years old. Between 1994 and 2006, a total of 9333 samples were processed. The sex distribution of the patients and the aetiology of burns are presented in Tables 1 and 2. It is interesting to note that in our series, male children outnumbered females by 13.2%. Mortality figures are presented in Table 3. This study focuses exclusively on the microbiological profile and no attempt has been made to correlate this with clinical data. We desire to do this as a separate study.

**Table 1:** Age sex distribution age - 1 month - 15 years

*Year*	*1994*		*1995*		*1996*		*1997*		*1998*		*1999*		*2000*		*2001*		*2002*		*2003*		*2004*		*2005*		*2006*		*Total*		
No. of Patients	112		129		153		144		157		132		135		122		92		96		77		73		112		1534		
														
	No.	%	No.	%	No.	%	No.	%	No.	%	No.	%	No.	%	No.	%	No.	%	No.	%	No.	%	No.	%	No.	%	*Tested for	NO.	%
Male	58	51.8	75	58.1	84	54.9	70	48.6	92	58.6	80	60.6	81	60.0	72	59.0	56	60.9	58	60.4	44	57.1	42	57.5	50	44.6	1534	868	56.6
Female	54	48.2	54	41.9	69	45.1	74	51.4	65	41.4	52	39.4	54	40.0	50	41.0	36	39.1	38	39.6	33	42.9	31	42.5	62	53.9	1534	666	43.4

**Table 2:** Aetiology

*Year*	*1994*		*1995*		*1996*		*1997*		*1998*		*1999*		*2000*		*2001*		*2002*		*2003*		*2004*		*2005*		*2006*		*Total*		
Total	112		129		153		144		157		132		135		122		92		96		77		73		112		1534		
														
	No.	%	No.	%	No.	%	No.	%	No.	%	No.	%	No.	%	No.	%	No.	%	No.	%	No.	%	No.	%	No.	%	*Tested for	NO.	%
Scalds	82	73.2	98	76.0	105	68.6	108	75.0	124	79.0	100	75.8	83	61.5	96	78.7	65	70.7	75	78.1	62	80.5	56	76.7	94	83.9	1534	1152	75.1
Flame	15	13.4	18	13.9	25	16.4	22	15.3	15	9.6	20	15.1	32	23.7	18	14.8	14	15.2	14	14.6	8	10.3	9	12.3	9	8.0	1534	221	14.4

Crackers	3	2.7	3	2.3	16	10.4	4	2.8	5	3.2	4	3.0	7	5.2	0	0.0	2	2.2	3	3.1	0	0.0	1	1.4	4	3.6	1534	52	3.4

Contact	9	8.0	5	3.9	5	3.3	5	3.5	2	1.3	5	3.7	4	3.0	3	2.5	4	4.3	2	2.1	2	2.6	2	2.7	1	0.9	1534	49	3.2

Electrical	1	0.9	4	3.1	2	1.3	3	2.1	5	3.2	1	0.8	6	4.4	2	1.6	4	4.3	2	2.1	4	5.2	3	4.1	3	2.7	1534	40	2.6

Chemical	2	1.8	1	0.8	-	-	0	0.0	4	2.5	1	0.8	3	2.2	0	0.0	0	0.0	0	0.0	1	1.3	2	2.7	1	0.9	1534	15	1

Tar	0	0.0	0	0.0	0	0.0	1	0.7	2	1.3	1	0.8	0	0.0	0	0.0	0	0.0	0	0.0	0	0.0	0	0.0	0	0.0	1534	4	0.3

Frostbite	0	0.0	0	0.0	0	0.0	1	0.7	0	0.0	0	0.0	0	0.0	0	0.0	0	0.0	0	0.0	0	0.0	0	0.0	0	0.0	1534	1	0.1

**Table 3:** Mortality statistics

*Year*	*1994*		*1995*		*1996*		*1997*		*1998*		*1999*		*2000*		*2001*		*2002*		*2003*		*2004*		*2005*		*2006*		*Total*		
Total no. of Patients	112		129		153		144		157		132		135		122		92		96		77		73		115		1534		
													
	No.	%	No.	%	No.	%	No.	%	No.	%	No.	%	No.	%	No.	%	No.	%	No.	%	No.	%	No.	%	No.	%	*Tested for	No.	%
No. of Deaths	11	9.8	8	6.2	13	804	12	8.3	14	8.9	13	9.8	20	14.8	18	14.8	12	13.0	13	13.5	7	9.1	6	8.2	8	7.0	1534	155	10.1

### Wound treatment

Closed dressings using silver sulphadiazine ointment were used in all patients without exception. The burn wounds were washed daily to remove necrotic tissue and the remnants of the previous day's ointment.

### Procedure for wound sampling

Microbial colonization of all wounds was studied from the time of admission to discharge. On admission, the sampling procedure included swabs that were taken from clinically deep areas of the burn wound prior to any cleansing. Swabs were taken twice weekly. The bandages were removed, the remnants of the previous day's ointment were washed away and the wounds were swabbed and cultured as follows: A sterile cotton swab is moistened with sterile normal saline. This swab is rubbed onto the burn wound surface. Swabs are taken from areas which appear deep, areas with discharge, thick eschar, etc. The soabs are then sent for culture.

### Microbiology

The swabs are transported to the laboratory for processing immediately. They are streaked onto a differential medium (e.g.; Mac Conkey agar] and an enriched medium (e.g.; blood agar). Isolation is carried out by the conventional T-method using sterile nichrome loop. These plates are incubated at 37 °C for 16-18 h. The basic aim was to isolate the organisms predominant on the burn wound and determine their sensitivity to various antibiotics for clinical purposes.

Antibiotic sensitivity of isolates obtained from the burn wound was carried out by filter paper disc diffusion method (Kirby Bauer method). Sterile commercially available filter paper discs, onto which a definite amount of antibiotic has been absorbed, are used. Since the antibiotic in the disc tends to diffuse more onto the surface of the agar than into the deeper layers, the plate is surface spread with the organisms. A broth culture of the isolate is prepared using sterile peptone water comparable to 0.5 Macfarlands turbidity standard (i.e.1×10^7^ to 1×10^8^ organisms/ml). Approximately 0.2 ml of this broth culture is surface spread onto sterile Mac Conkey agar plate (for gram-negative organisms)/sterile blood agar plate (for gram-positive organisms), so as to get a matt growth.

**Figure 1:**
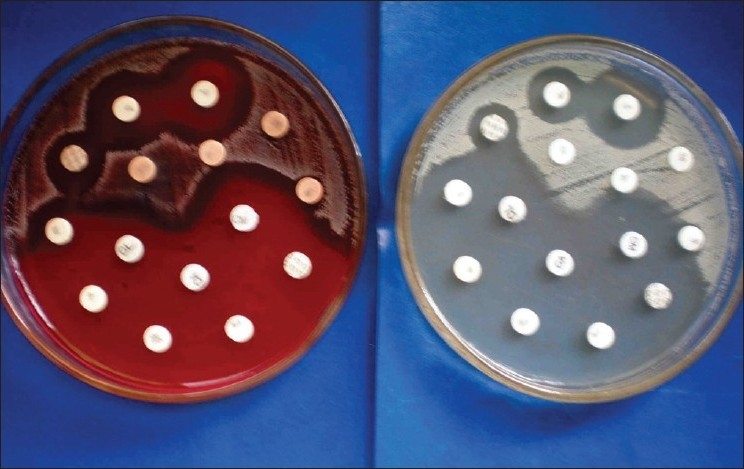
Comparitive study with mueller hinton agar and blood agar

**Figure 2:**
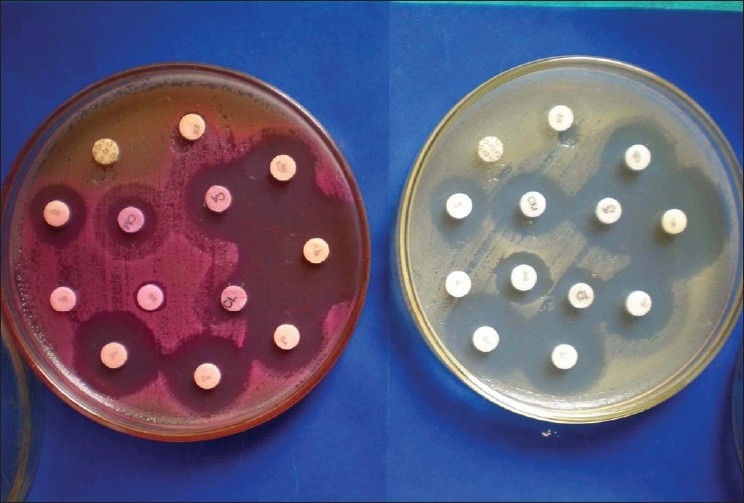
Comparitive study with mueller hinton and mac conkey agar

Sterile antibiotic discs are equidistantly placed on these plates and gently pressed onto the medium with the help of sterile forceps to ensure complete contact with the agar surface. The plates are incubated at 37° C for 16 to 18 h. Zones of inhibition are measured in millimetres and the organisms classified as sensitive or resistant according to the zone size interpretation chart. It must be noted that our antibiotic sensitivities were not carried out on Mueller Hinton agar as advocated by some authors[20,21]. Subsequently, we have carried out a comparative study and tested antibiotic sensitivities for 10 different burn wound isolates on Mueller Hinton and Blood agar/ Mac Conkey agar and found no significant difference in the [Figure 1 & 2] results.

## RESULTS

In the present study, a total of 9333 samples were processed from patients admitted to the burns unit; 1281 samples (13.72%) showed absence of bacterial pathogens [Table 4].

**Table 4:** Bacteriological studies

*Year*	*1994*		*1995*		*1996*		*1997*		*1998*		*1999*		*2000*		*2001*		*2002*		*2003*		*2004*		*2005*		*2006*		*Total*		
Patients	112		129		153		144		157		132		135		122		92		96		77		73		112		1534		
														
	No.	%	No.	%	No.	%	No.	%	No.	%	No.	%	No.	%	No.	%	No.	%	No.	%	No.	%	No.	%	No.	%	*Tested for	No.	%
Total	336		524		782		1184		1244		1080		949		879		472		519		422		383		559		1534	9333	
No	58	17.3	74	14.1	175	22.4	202	17.1	205	16.5	107	9.9	125	13.2	105	11.9	60	12.7	31	6.0	29	6.9	47	12.3	63	11.3	1534	1281	13.7
Growth																													
Growth	278	82.7	450	85.9	607	77.6	982	82.9	1039	83.5	973	90.1	824	86.8	774	88.1	412	87.3	488	94.0	393	93.1	336	87.7	496	88.7	1534	8052	86.3
Isolates	379		696		1016		1931		2059		1557		1530		1362		689		972		805		509		634		1534		

The most common isolate was *Klebsiella* (33.91%) followed by *Pseudomonas aeruginosa* (31.84%). A detailed break-up is given in Table 5.

**Table 5:** Organisms isolated

*Year*	*1994*		*1995*		*1996*		*1997*		*1998*		*1999*		*2000*		*2001*		*2002*		*2003*		*2004*		*2005*		*2006*		*Total*		
Total	379		696		1016		1931		2059		1557		1530		1362		689		972		805		509		634		14131		
Isolates																													
Organisms	No	%	No	%	No	%	No	%	No	%	No	%	No	%	No.	%	No.	%	No.	%	No.	%	No.	%	No.	%	*Tested for	No.	%
*Klebsiella*	176	46.4	293	42.1	449	44.2	671	34.7	695	33.8	561	36.0	560	36.6	456	33.5	233	33.8	312	32.1	211	26.2	103	20.2	69	10.9	14131	4786	33.9
*Pseudomonas*	146	38.5	258	37.1	307	30.2	444	23.0	432	21.0	355	22.8	506	33.1	472	34.7	219	31.8	365	37.6	285	35.4	285	56.0	422	66.6	14131	4494	31.8
*Acinetobacter*	15	4.0	1	0.2	-	-	0	-	0	-	0	-	0	-	0	0.0	0	0.0	0	0.0	0	0.0	0	0.0	0	0.0	14131	16	0.1
*E. Coli*	-	-	19	2.7	67	6.6	349	18.1	265	12.9	147	9.4	113	7.4	141	10.4	121	17.6	217	22.3	227	28.2	81	15.9	79	12.5	14131	1826	12.9
*Proteus*	-	-	3	0.4	20	2.0	2	0.1	50	2.4	42	2.7	51	3.3	20	1.5	5	0.7	11	1.1	18	2.2	5	1.0	1	0.2	14131	228	1.6
*S. aureus*	33	8.7	115	16.5	173	17.0	465	24.1	539	26.2	415	26.7	280	18.3	248	18.2	101	14.7	59	6.1	55	6.8	35	6.9	58	9.1	14131	2576	18.2
*S. albus*	9	2.4	4	0.6	-	-	0	-	0	-	0	-	0	-	0	0.0	4	0.6	0	0.0	0	0.0	0	0.0	1	0.2	14131	18	0.1
Streptococcus	7	-	3	0.4	-	-	0	-	78	3.8	37	2.4	20	1.3	25	1.8	6	0.9	5	0.5	3	0.4	0	0.0	1	0.2	14131	175	1.2
Yeast																			3	0.3	6	0.7			3	0.5	14131	12	0.1

A detailed analysis on individual microorganisms and their antibiotic sensitivities, along with changing trends over this 13-year period is presented. What follows is a bird's eye view of the microorganism and its dominant sensitivity pattern.

*Klebsiella* was sensitive to Gatifloxacin (86.3%)

Cefaperazone+Sulbactam (82.8%)

Piperacillin+Tazobactam (77.4%)

Meropenem (72.4%)

Amikacin (66.9%)

Azithromycin (60.4%).

*Pseudomonas* was sensitive to Cefoperazone+Sulbactam (73.9%)

Piperacillin+Tazobactam (72.2%)

Amikacin (62.3%)

Azithromycin (56.3%)

Meropenem (55.8%)

Gatifloxacin (49.9%).

*S. aureus* was sensitive to Sparfloxacin (90.4%)

Cefpirome (80.9%)

Piperacillin+Tazobactum (78.4%)

Netilmicin (77.2%)

Imipenem (64%)

Erythromycin (51.1%).

*E. coli* was sensitive to Ticarcillin+Clavulanic acid (67.2%)

Meropenem (63.6%)

Amikacin (42.7%)

Azithromycin (27.4%)

Gatifloxacin (61.9%)

Cefoperazone+sulbactam (69.1%).

*Proteus* was sensitive to Piperacillin+Tazobactam (97.1%)

Meropenem (82.9%)

Ceftrioxone and Ceftizoxime (64.6%)

Gatifloxacin (62.9%)

Amikacin (55.8%)

Azithromycin (47.8%).

## DISCUSSION

Thermal injury destroys the barrier function of skin, allowing microbial colonization of wounds and even with the use of topical antimicrobials, contamination of wounds is unavoidable.

The type and amount of microorganisms on and in the injured tissue influence wound healing,[7] the frequency of invasive infection and the clinical characteristics of such infections as well as the risk of dissemination. Thus, knowledge of the burn ward microbial flora and the current antibiotic sensitivities at any time is important for the clinician treating burn sepsis.

It has been our observation that when patients are brought to the hospital with exposed burnt areas, the initial swabs reveal no growth. After applying a closed dressing, repeat swabs from the same patient reveal presence of microorganisms. Admittedly, burn biopsy is a better tool to determine microbiological colonization and invasion and for quantitative evaluation. It is also less fallacious. Many centres however, in our country and the world over as well, continue to rely on swab culture reports to initiate treatment as the specimens are easier to obtain and processing time comparatively lesser.

In this study, we found that the most frequent isolates were *Klebsiella* followed by *Pseudomonas* (31.84%). Compared to several earlier reports on burn wound colonization and invasive infection, one of the most striking differences is the frequency of *Klebsiella* in this study, which is contrary to findings in other studies in which Klebsiella formed a small number of total isolates.[1,8–12] It was interesting to note that two units in Nigeria[13,14] also had *Klebsiella* as the most frequent pathogen isolated. The burns unit at Ain Shans University Hospital, Egypt, reported *Klebsiella* as the second most common organism in their study.[19]

The pattern of bacterial sensitivities is subject to frequent changes. Its assessment is important for clinical and epidemiological purposes.

For ease of discussion, the various antibiotics were grouped under their respective generic families, e.g.; penicillins, macrolides, quinolones. Antibiotics which did not fit in were placed in the “other antibiotics” group.

Data mining revealed that while *Klebsiella* was the dominant organism from 1994 to 2000, *Pseudomonas* gained the upper hand from 2001. *Klebsiella* was the dominant organism in 2002; subsequently, *Pseudomonas* became the reigning *organism* from 2003 to 2006 (66.6% in 2006 while the percentage of *Klebsiella* is 10.9%). It will be a revelation to us too to see how this pans out. The prevalence of *Escherichia* was on the rise from 2001 to 2004 and it is starting to wean off from 2005 15.9% and 12.5% (2006) in successive years. The percentage incidence of *Staphylococci* is on the decline from 2002 to 2005.

## CONCLUSION

It may be concluded that the composition of bacterial flora in burns is dependent not only on the depth and extent of the burn but also on the site of burn, the duration of burn, the age of the patient and his/her co-morbidities.[15] Burn wound monitoring requires the study of changing bacterial flora and the antibiotic sensitivity reports. Repeated swab cultures and antibiograms are advised for proper selection of antibiotics to control sepsis.[18] The development of resistance to a particular antibiotic is dependent on the use of that antibiotic in society at large. Overuse of any antibiotic predisposes to development of resistance. Our unit gets patients from all over Mumbai, other parts of the state of Maharashtra and at times, from other states too. Due to this huge diversity, we have a particular microorganism predominant at a particular point in time, but then, it is also difficult to comment on the source of the changing trends.
